# Optimizing Wireless Connectivity: A Deep Neural Network-Based Handover Approach for Hybrid LiFi and WiFi Networks

**DOI:** 10.3390/s24072021

**Published:** 2024-03-22

**Authors:** Mohammad Usman Ali Khan, Mohammad Inayatullah Babar, Saeed Ur Rehman, Dan Komosny, Peter Han Joo Chong

**Affiliations:** 1 Department of Electrical Engineering, University of Engineering and Technology, Peshawar 25120, Pakistan; musmank@gmail.com (M.U.A.K.); babar@uetpeshawar.edu.pk (M.I.B.); 2 College of Science and Engineering, Flinders University, Adelaide, SA 5042, Australia; 3 Department of Telecommunications, Brno University of Technology, 601 90 Brno, Czech Republic; komosny@vut.cz; 4 Department of Electrical and Electronic Engineering, Auckland University of Technology (AUT), Auckland 1010, New Zealand; peter.chong@aut.ac.nz

**Keywords:** light fidelity, WiFi, handover, DNN, HLWNet

## Abstract

A Hybrid LiFi and WiFi network (HLWNet) integrates the rapid data transmission capabilities of Light Fidelity (LiFi) with the extensive connectivity provided by Wireless Fidelity (WiFi), resulting in significant benefits for wireless data transmissions in the designated area. However, the challenge of decision-making during the handover process in HLWNet is made more complex due to the specific characteristics of electromagnetic signals’ line-of-sight transmission, resulting in a greater level of intricacy compared to previous heterogeneous networks. This research work addresses the problem of handover decisions in the Hybrid LiFi and WiFi networks and treats it as a binary classification problem. Consequently, it proposes a handover method based on a deep neural network (DNN). The comprehensive handover scheme incorporates two sets of neural networks (ANN and DNN) that utilize input factors such as channel quality and the mobility of users to enable informed decisions during handovers. Following training with labeled datasets, the neural-network-based handover approach achieves an accuracy rate exceeding 95%. A comparative analysis of the proposed scheme against the benchmark reveals that the proposed method considerably increases user throughput by approximately 18.58% to 38.5% while reducing the handover rate by approximately 55.21% to 67.15% compared to the benchmark artificial neural network (ANN); moreover, the proposed method demonstrates robustness in the face of variations in user mobility and channel conditions.

## 1. Introduction

Despite the successful global standardization and commercialization of the 5G wireless communication network, its current performance, as of 2020, is anticipated to be inadequate to meet future demands. This is primarily attributed to the rapid development of emerging technologies, for example, the IoT (Internet of Things) and VBR (Virtual Mobile Reality) [1,2]. Moreover, there is a significant surge in the need for mobile traffic, with a sevenfold growth from 2017 to 2022, reaching around 77.5 Exabyte each month [3]. In contrast to 5G, the upcoming wireless networks, commonly referred to as the 6G wireless networks, are anticipated to prioritize human-centric features. It aims to provide a high data rate, enhanced security, improved energy, and cost competence [4]. In the context of evolving telecommunications networks, the future landscape—encompassing the Internet of Everything (IoE) and the seamless connection of people, processes, things, data, and all elements—necessitates uninterrupted Internet connectivity at all times [5].

To maintain signal continuity while improving user mobility, a highly promising idea that has attracted considerable research attention is the notion of Hybrid Light Fidelity (LiFi) and Wireless Fidelity (WiFi) networks, referred to as HLWNets [6]. These networks are being explored as potential elements for inclusion in the 6G network [7]. Notably, in indoor settings, Hybrid Light Fidelity and Wireless Fidelity demonstrate clear benefits over traditional networks such as RF-Heterogeneous [8,9], including enhanced data transfer rates, minimized interference, energy efficiency, and seamless connectivity. In contrast, a LiFi network, utilizing light-emitting diodes (LEDs), operates within a broader, untapped, and unlicensed spectrum of 300 THz compared to radio waves [10,11]. A LiFi network can achieve exceptionally high data transmission rates with notable power effectiveness, ease of availability, cost-effectiveness, minimal interferences, and robust security [5,8]. However, LiFi networks do have their constraints. A standalone LiFi network only covers a small area, so users cannot move around freely without disruption in communication [12]. Furthermore, the susceptibility of line-of-sight (LoS) links among LiFi access points (APs) and users makes them vulnerable to disruption from possible shadowing and angular misalignments [13]. Similarly, WiFi can act as an alternative to LiFi in scenarios where optical signals are unreachable, ensuring consistent connectivity and user mobility [14], with several benefits over WiFi such as efficient use of the spectrum, improved indoor localization, and immunity to radio frequency restrictions. Conversely, because of the extremely dense availability of LiFi access points and the susceptibility of optical light to LoS propagation, the changeover procedure for choosing between LiFi and WiFi becomes increasingly crucial compared to conventional heterogeneous networks [15].

**Figure 1:**
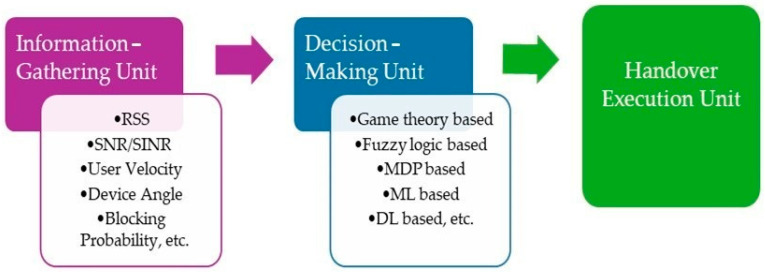
Handover framework.

Based on an earlier investigation outlined in reference [16], a typical handover framework comprises three distinct units: the information-gathering unit (IGU), the decision-making unit (DMU), and the handover execution unit (HEU). Figure 1 shows the interaction of these units.

The IGU plays a crucial role in gathering essential information for the process of handover decisions. It intermittently transmits this data to the decision-making unit, containing an algorithm for the handover. Subsequently, the DMU determines the most appropriate access network and measures the need for the handover. An actual handover triggers when the HEU takes a request for handover from the DMU. The handover framework contains a handover algorithm dictating the rules for decision-making. From a mathematical standpoint, this algorithm is comparable to a function of multiple inputs and a single output. The function takes inputs from entire metrics utilized in a decision process, and the outputs are expressed as “0” or “1”, indicating the outcomes of the decisions. Various measured tools, such as fuzzy logic [17,18], Markov decision [19,20], and game theory [21,22] have been utilized in developing handover algorithms for heterogeneous radio frequency networks. In research [23], the authors present a unique perspective by approaching the handover challenge in Hybrid LiFi and WiFi networks equally to a task of pattern recognition, expressing this as a problem of binary classification. Both logistic regression and a support vector machine were employed in formulating these handover algorithms. While the results obtained from the simulations in the experiments indicate that logistic regression-based and support vector machine-based handover models outperform previous models, improving handover accuracy further is still difficult due to the inherent limitation of logistic regression and support vector machines in addressing non-linear boundary classification issues. deep neural networks (DNNs) possess the capability to autonomously acquire pertinent features from raw data, thereby diminishing the necessity for manual feature extraction and selection. DNNs can model highly non-linear relationships between inputs and outputs, which might be difficult for traditional machine learning algorithms like linear regression or SVMs. Although traditional machine learning models are more interpretable, ongoing endeavors aim to enhance the interpretability of deep neural networks (DNNs) through methods like attention mechanisms and model visualization. To improve the handover decision accuracy and enhance various performance indicators, we investigate the application of neural networks (NNs) in the context of a Hybrid LiFi and WiFi network. NNs are recognized for their ability to handle complex, non-linear issues without relying on explicit models, as demonstrated in previous studies [24,25,26]. Previous efforts have sought to integrate NNs for managing the handovers in heterogeneous networks.

In study [15], aimed at boosting mobile user throughput, a NN was employed for regulating the prediction for selecting either LiFi or WiFi networks. Although the research demonstrated the effectiveness of neural networks in designing handover algorithms, certain gaps persist. Firstly, complex issues that could be covered for users—for example, the orientation of the devices—were overlooked in the decision process. Additionally, the NN-based algorithms described in [15] do not directly determine whether to execute a handover based on the given inputs; instead, they provide a network preference score, requiring additional steps for a comprehensive handover decision. To address these issues, we propose an innovative NN-based handover approach, treating the handover problem within a Hybrid LiFi and WiFi network as a binary classification task. Our method in this study involves a DNN for deciding upon a handoff from LiFi to WiFi and vice versa. A large portion of the feature extraction process is automated by deep learning using an SDN-enabled controller, which reduces the need for manual human intervention. Additionally, it facilitates the utilization of extensive datasets, which makes it an ideal method for a scalable real-world scenario. In contrast to the handover approach outlined in [15], the method used in this study offers the ability to integrate multiple attributes and the orientation of devices as input features to the neural network, enabling timely and accurate decisions in addition to a streamlined handover process. As the handover process is treated as binary classification, the approach makes handover decisions directly based on a given set of inputs. Simulation findings indicate that the suggested method achieves a handover accuracy of over 95%, nearly optimum user throughput, and an important reduction in handover rates as compared to the established benchmarks (such as SVM and ANN); furthermore, the proposed approach exhibits better resilience under varying operational conditions.

The subsequent sections of this document are organized in the following manner. Section 2 presents the HLWNet model. In Section 3, a DNN-based handover scheme is presented. Section 4 offers a detailed presentation of the simulation results along with relevant discussions. Lastly, Section 5 presents the final observations.

## 2. System Model

**Figure 2:**
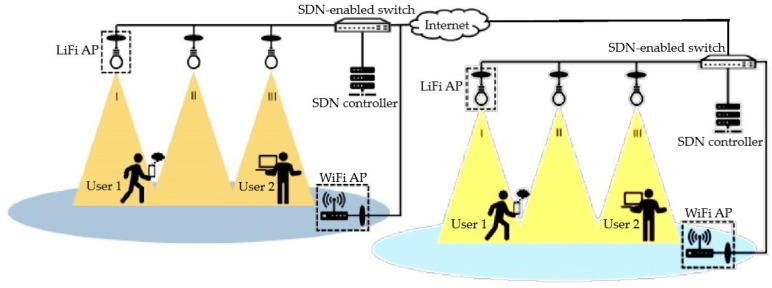
An SDN-enabled Hybrid LiFi and WiFi network structure.

This paper employs a Hybrid WiFi–LiFi scheme, which contains many LiFi access points and a single WiFi access point. To prevent interference with the LiFi downlink transmissions, a bidirectional LiFi network is implemented, where visible light and infrared are used for downlink and uplink communications [27]. Every cell of LiFi is composed of vertically oriented LEDs and infrared photodetectors as transceivers. Managing LiFi and WiFi access points owned by the same entity is viable through a centralized control unit. The controller helps in attaining efficient routing and resource allocation at the network level [14]. This framework relies on Software-Defined Networking (SDN), which separates the data plane from the control plane of the forwarding devices. Additionally, each WiFi and LiFi access point is connected to an SDN-enabled switch. This switch retrieves key performance indicator information from these access points via SDN agents. The SDN controller oversees the routing of data packets to each access point, as depicted in Figure 2. For multiple access, a Time Division Multiple Access (TDMA) is engaged in all LiFi cells, while WiFi utilizes Carrier-Sense Multiple Access with Collision Avoidance (CSMA/CA) as its Medium Access Control (MAC) protocol [28]. The structure’s model includes the LiFi/WiFi channel model, realizable data rates for LiFi/WiFi, orientation-based random waypoints, and considerations for light-path obstruction, which aligns with the previous work in [16]. The variable notations are consistent with those introduced in [16].

## 3. Neural Network-Based Handover Method

This section presents the handover approach utilizing a feedforward neural network (FNN), where the handover process is treated as a problem of binary classification. The Intelligent Gateway Unit (IGU) in Figure 1—located in the user equipment—gathers information periodically, including the quality of the current and target channels, light-path obstruction, mobility of users, and the orientation of devices. Given that our approach exclusively addresses vertical handover (VHO), the IGU focuses on monitoring the quality of the channel associated with the access point of LiFi with maximum Signal-to-Interference plus Noise Ratio (SINR) and WiFi access point [16]. Information such as the SINR/SNR [15,29] is accessible to mobile nodes, while details of velocity and angularity associated with the user are obtained through an IMU (Inertial Measurement Unit) [30]. Moreover, an average duration of light-path availability and unavailability is detailed and informed to compute an interruption estimation and a rate of recovery, denoted as β_1_ and β_2_ [17]. The collected data are then consolidated into an input vector to train the neural network. Subsequently, all inputs undergo transformation via several hidden layers containing several neurons, where a handover decision is finally made by the neural network. In the proposed system, a binary “1” indicates an immediate execution of a handover, while a binary “0” implies no execution of a handover. If transitioning from WiFi to LiFi and from LiFi to WiFi, two distinct neural networks must be trained. The subsequent section will provide detailed explanations of the neural network architecture, datasets, and training/testing processes.

### 3.1. Network Architecture

**Figure 3:**
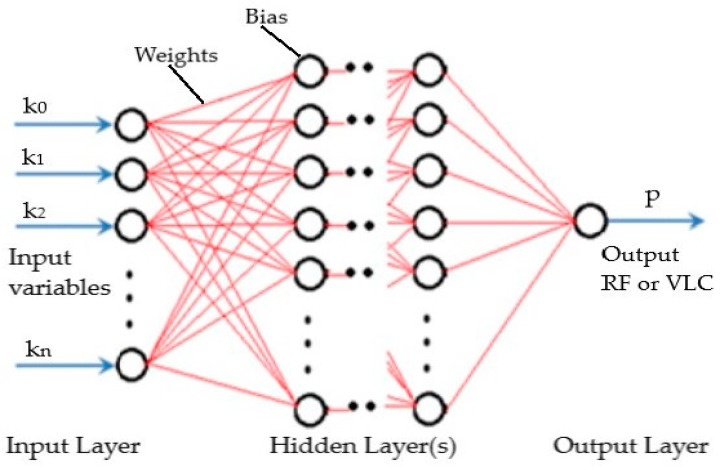
Trained feedforward deep neural network (FDNN) architecture.

The handover method, discussed in Section 2, can be thought of as a non-linear function, denoted as f(k), yielding a singular output represented by a “1” or “0”. In this context, k represents one of the elements in the input vector Rn, which consists of n different features. Hence, the process of developing the handover method entails determining the most suitable hypothesis or approximator Ӆ for the function f(k). Utilizing a neural network, specifically a feedforward neural network composed of interconnected neurons, offers a promising solution. Neural networks, particularly those with a hidden layer and suitable activation functions, have the capability to approximate any continuous function [31]. To tackle this challenge, the article employs a trained L-layer feedforward deep neural network (FDNN) architecture, as depicted in Figure 3. In this architecture, there is complete pairwise connectivity between neurons in sequential layers. Every neuron inside this structure experiences a procedure in which the total of weighted outputs from the preceding layer, in addition to a bias, is sent into an activation function. This activation function imparts non-linearity to the neuron. The computational process—which begins at the input layer, progresses through the hidden layers, and ends at the output layer—is known as forward propagation. Through incorporating a maximum likelihood estimation (MLE) threshold, this method allows for the identification of an approximator function that produces either a “1” or “0” as its output. The forward propagation is expressed:

(1) $$\alpha^{\left(1\right)}=f(\psi^{\left(1\right)}\left[\begin{array}{c} k_{0}\\ K \end{array}\right])$$

(2) $$\alpha^{\left(i+1\right)}=f(\psi^{\left(i\right)}\left[\begin{array}{c} \alpha_{0}^{\left(i\right)}\\ \alpha^{\left(i\right)} \end{array}\right])$$

(3) $$Ӆ\left(k;\;\psi \right)=f(\psi^{\left(D\right)}\left[\begin{array}{c} \alpha_{0}^{\left(D\right)}\\ \alpha^{\left(D\right)} \end{array}\right])$$

where $\psi^{\left(i\right)}\epsilon \;R^{N\left(i+1\right)\;X\;\left(\mathrm{Ni}+1\right)}$ and *D* represent the function controlling the matrix of weights and the depth of the DNN, respectively. *N* and *α* represent the number and activation of neurons, respectively. Input bias is given as *k*_0_ and *α*_0_. The mapping from the first to the last layer is controlled by the activation function ‘*q*’:

(4) $$q\left(k\right)\frac{1}{1+e^{-k}}\;\in {\left(0,\;1\right)}^{n}$$

Given that the hypothesis Ӆ(k; ψ) produces an estimated probability *p* = 1 for input *k*, the maximum likelihood estimation (MLE) is able to classify it as either “0” or “1”, given as the following; in addition, *n*_(*i*+1)_ *x* (*n*_*i*_+1):

(5) $$p=\{\begin{array}{c} 1\;if\;Ӆ\geq 0.5\\ 0\;if\;Ӆ<0.5 \end{array}$$

Hence, when a given input vector *k* is processed by an FDNN with optimized parameters, the decision-making unit will initiate a handover to the handover execution unit when the FDNN output is 1; alternatively, the user equipment remains connected to the same network.

### 3.2. Dataset Generation

Sufficient quantities of data with precisely annotated real-world information are required for activities that include supervised learning. Nevertheless, existing research on neural handover algorithms lacks comprehensive details on generating labeled data [15,32] to the best of our knowledge. Unlike other classification challenges such as object detection, determining the target value for handover execution based on a given set of inputs is not straightforward. It remains uncertain whether the handover process is justified, even including data regarding the quality of the channel, the mobility of the user, and the frequencies of interruption and recovery. In order to create datasets with labels, we suggest a novel method that involves the following procedures. At first, we generate sequences of tuples that represent dynamic information, such as parameters like velocity, direction, position, and polar angle. The revised direction-dependent Random Waypoint model described in Section 2 of the cited article [16] is used to accomplish this. Afterward, we calculate the Signal-to-Interference plus Noise Ratio (SINR) and Signal-to-Noise Ratio (SNR) data for individual users on all the LiFi and Wifi channels at every instance. The calculations rely on data regarding the velocity, direction, position, and polar angle, as described in the simulation scenarios presented in subsections 2A and 2B of the same source [16]. In order to replicate the obstruction of a light channel, we utilize an ON–OFF model that includes designated rates for interruption and recovery, represented as 1 and 2. To simplify matters, we assume that the Signal-to-Interference plus Noise Ratio (SINR) value of the LiFi channel is 0 while the optical channel is blocked. By following these processes, we acquired the dataset, which was subsequently partitioned into training (70%), test (15%), and validation (15%) sets for the purpose of evaluating performance.

### 3.3. Training and Testing

This paper generates the sample dataset using the approach described in [33]. Once the FDNN architecture is established, a hypothesis Ӆ(*k*) is derived. The next step involves model training to fine-tune hyperparameters. The assessment of hypothesis performance is conducted using data loss, measuring compatibility between predictions and the ground truth. This transforms the FDNN training into an optimization problem solved through the gradient descent algorithm in [34]. The training process also includes tuning the FDNN model through cross-validation. The testing process involves evaluating two aspects: the trained model and the wireless network. Accuracy measures how well the trained model matches access point selection results to ground-truth labels, while achievable throughput directly assesses network performance. The focus lies in analyzing the throughput gap between the trained model and the ground truth label. Ultimately, the hypothesis’s performance is evaluated on the test set by examining average loss plots and confusion matrices [35].

### 3.4. Simulation

**Figure 4:**
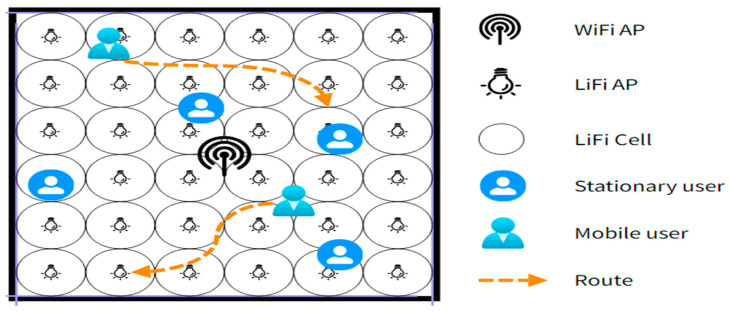
Simulation scenario of Hybrid LiFi and WiFi network.

The default simulation scenario, exhibited in Figure 4, features an enclosed workspace with measurements of (18 × 18 × 3) cubic meters. In this environment, there is a single 2.4 GHz WiFi access point, which provides coverage to the whole room, and a lattice topology comprising 36 LED-based LiFi access points (APs). The coverage of each LiFi AP is confined to a smaller area, known as an attocell. For the sake of simplicity, we have considered the constant reflectivity model for LiFi, while no interference is counted in the WiFi system. The FDDN model contains three fully connected layers, which include a solitary hidden layer of 10 neurons and a bias. To begin, we assess the most effective training interval for the dataset to aid the decision-making process of the FDNN responsible for transitioning between LiFi and WiFi. Assuming the user equipment is presently connected to the LiFi network, we choose distinct values for the interval τ in order to label 10,000 sets of data. Subsequently, the neural network is trained using these labeled datasets. Afterward, the model that is trained is utilized to replicate the mean throughput of 100 consumers during a 300-s timeframe. Monte Carlo simulations are conducted to evaluate the performance of the proposed learning model. To evaluate the efficiency of the suggested neural handover scheme, we utilize kernelized SVM (K-SVM), kernelized LR (K-LR), and ANN-based algorithms for the handover as benchmarks [15]. The suggested deep neural network (FDNN) is subsequently evaluated against these reference standards, utilizing the same simulation parameters. To assess the effectiveness of the handover strategy, we calculate the handover burden as a normal distribution with a 400-ms mean value [17]. The simulation involves, overall, 300 users accessing the model at separate times, each characterized by a uniformly distributed velocity magnitude (ranging from 0 to 3 m/s) and direction (ranging from 0 to 2π). The simulation duration for each user is set at 300 s. We can find simulation parameters in Table 1 and for other LiFi/WiFi parameters refer to [36].

## 4. Results and Discussion

**Figure 5:**
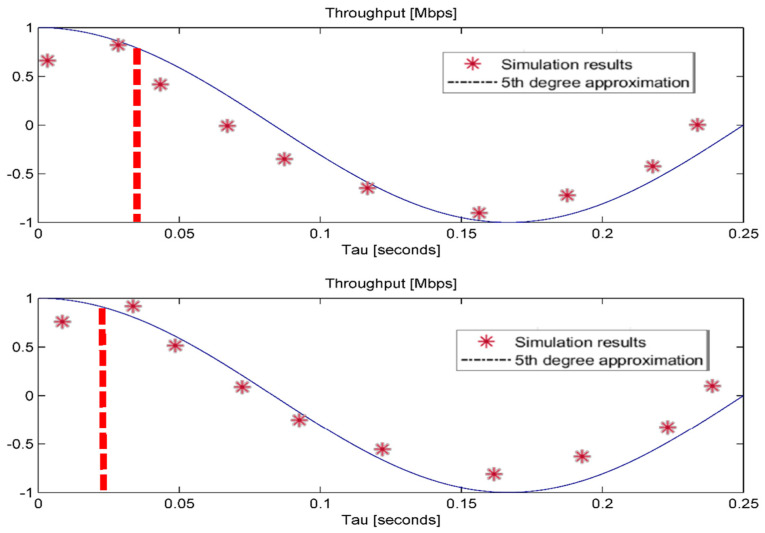
Performance using FDNN-based handover scheme (**Upper Panel**) WiFi to LiFi and (**Bottom Panel**) LiFi to WiFi. Axes are normalized.

Figure 5 illustrates an examination of the mean user throughput in relation to various τ values, utilizing fifth-degree polynomials to estimate their correlation. The results indicate a significant enhancement in average user throughput under the suggested feedforward deep neural network (FDNN)-based handover mechanism, reaching its highest point at around τ = 1.9 s. After this peak, throughput decreases rapidly as τ increases, stabilizing at approximately 25 Mbps. This observed trend is attributed to the effectiveness of the measured inputs in predicting the “near future” rather than long-term outcomes. The bell-shaped curve in Figure 5 emphasizes the viability of using a neural network for the purpose of developing a handover method. Failure to do so would result in a horizontal curve. Using a similar methodology, the most advantageous τ value for training the dataset to enhance the artificial neural network’s decision-making procedure for switching from WiFi to LiFi is determined to be near 2.1 s, which is depicted in Figure 5. As a result, the decision-making FDNN is trained using two datasets annotated with the optimal values τ = 1.9 s and τ = 2.1 s.

### 4.1. Handover Accuracy

**Figure 6:**
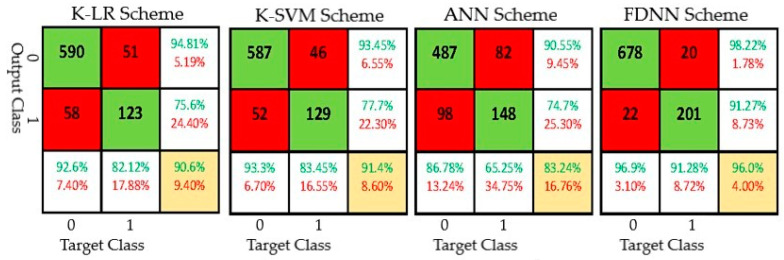
Handover accuracies of various schemes.

For the evaluation of handover accuracy, a total of 800 labeled datasets were randomly chosen from the test set, encompassing four distinct approaches. The accuracy results are shown in the confusion matrix in Figure 6. The findings indicate that the proposed handover method, which relies on FDNN, attains the best level of accuracy at 96%, accompanied by an F1 score of 0.91. Furthermore, the handover accuracies of K-SVM and K-LR are comparable, which aligns with the findings of a prior investigation [24]. Despite both K-LR (90.6%) and K-SVM (91.4%) methods achieving handover accuracy above 90%, their respective F1 scores are 0.79 and 0.81, which are inferior to the suggested FDNN-based approach. It is worth noting that the numerical outcomes for K-LR and K-SVM in this study differ slightly from those in [24] due to variations in the test sets used. The benchmark artificial neural network (ANN) exhibits the lowest accuracies in handover at 83.24% and achieves the least favorable F1 score of 0.70. The handover accuracy and F1 score of our scheme compared against the baseline methods are summarized in Table 2.

### 4.2. User Throughput and QoS

**Figure 7:**
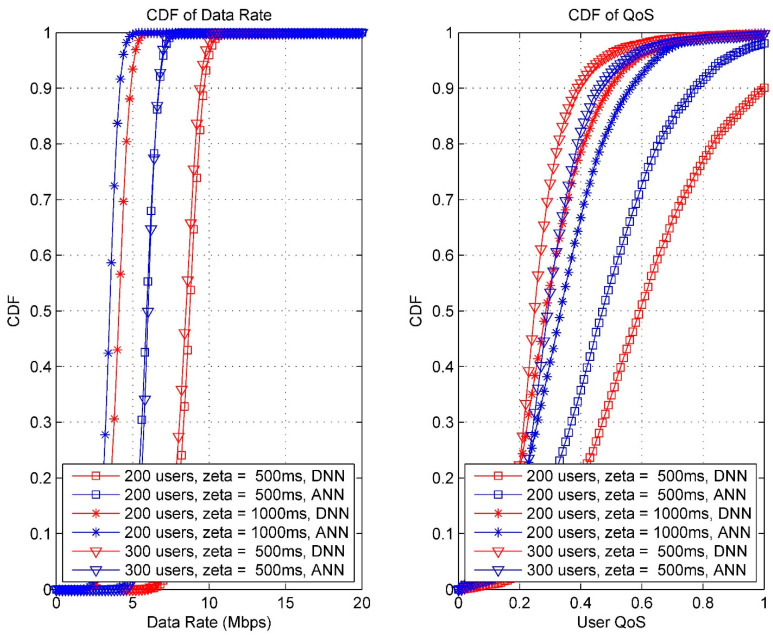
CDF curves for data rates and QoS (DNN vs. ANN).

**Figure 8:**
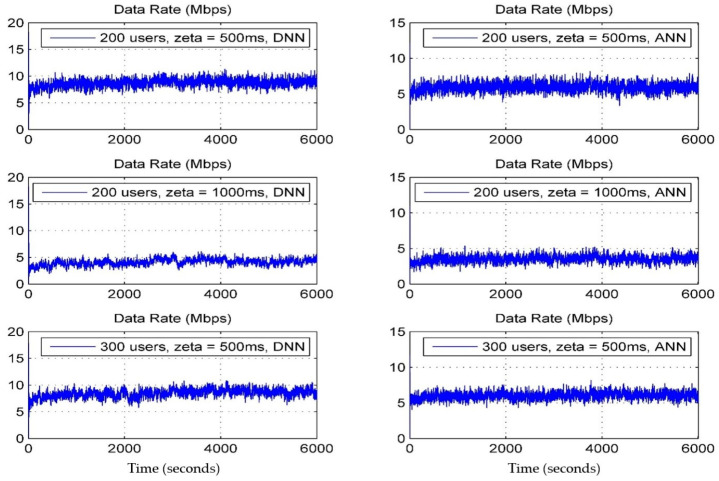
Data rates (DNN vs. ANN).

**Figure 9:**
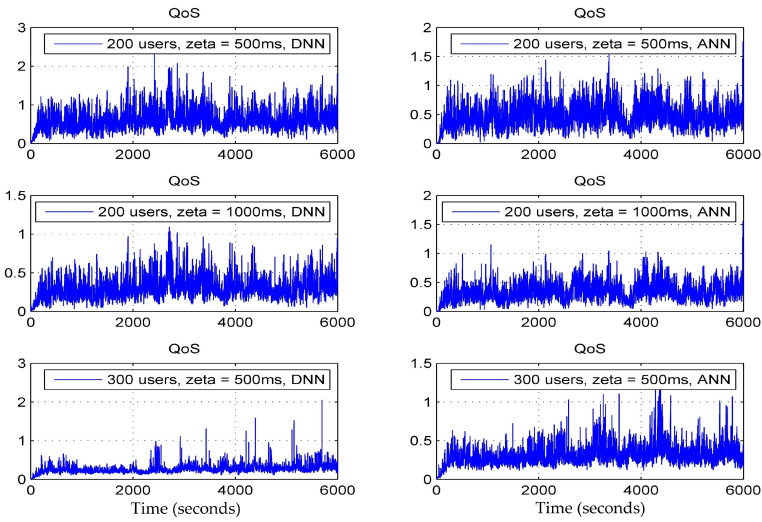
Quality of Service (DNN vs. ANN).

Figure 7 depicts a cumulative distribution function (CDF) chart, which showcases the average user throughput where zeta is the average handover overhead, in two distinct scenarios: the CDF of the data rate and the CDF of the quality of service (QoS). Here, the QoS is defined as the ratio of the data rate achieved by the user to the data rate required by that user. It is evident that, when utilizing a deep neural network (DNN), the user throughput consistently outperforms that of an artificial neural network (ANN) across various users. This suggests a significant enhancement in the received signal strength of user equipment (UE) with the DNN-based approach. Moreover, there is an improvement in the QoS with the DNN-based approach. The DNN-based method consistently achieves the highest user throughput, showing an enhancement ranging from approximately 18.58% to 38.5% compared to the benchmark ANN; furthermore, the CDF curves of the DNN-based approach for different user quantities exhibit greater variability, indicating superior robustness performance compared to the benchmark ANN. This superiority is attributed to our approach, which considers multiple attributes for making handover decisions. In Figure 8 and Figure 9, data rates (Mbps) and the QoS for various users are compared between the DNN and ANN approaches. The DNN-based technique exhibits lower average handover rates compared to the benchmark ANN, as illustrated in Figure 7. Specifically, the DNN-based approach reduces handover rates by approximately 55.21% compared to the ANN (67.15%).

### 4.3. Reliability

**Figure 10:**
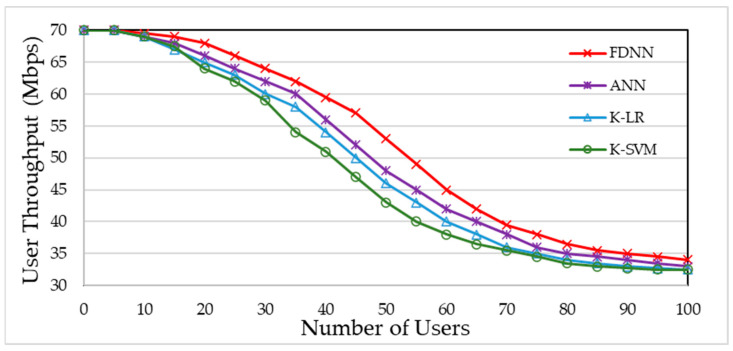
User throughput vs. number of users.

An effective handover algorithm should not only enhance the network throughput but also demonstrate adaptability to various network scenarios. In essence, it should exhibit resilience in the face of different operational conditions. We tested the robustness of our proposed scheme against different algorithms by assessing how the number of users and user velocity impact the throughput for each user. Figure 10 shows users’ throughput as a function of the number of users. As shown, the proposed FDDN method outperforms other benchmarks in most situations. It is crucial to emphasize that as the user count rises, network performance reaches a saturation point, limiting the effectiveness of various load-balancing methods in generating additional gains.

**Figure 11:**
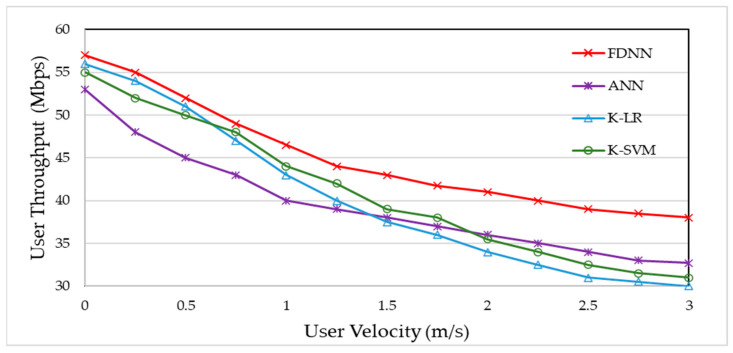
User throughput vs. user velocity.

Figure 11 illustrates user throughput in relation to user velocity. It indicates a decline in data rates for all methods with increasing user velocity. Notably, the proposed FDDN approach exhibits comparatively smaller variations, attributed to the consideration of user velocity in network selection. Our proposed algorithm consistently achieves the highest user throughput across various velocities, demonstrating greater resilience to speed variations compared to other benchmarks.

An FDNN-based handover method for HLWNets must navigate trade-offs such as balancing accuracy with latency and complexity, ensuring robustness without overfitting, and optimizing energy efficiency versus performance. By carefully managing these trade-offs, an effective handover method can be created.

## 5. Conclusions

A hybrid network that combines LiFi and WiFi, referred to as HLWNet, offers substantial advantages in the domain of wireless data transmissions. This study addresses the topic of handover decision-making in HLWNet by treating it as a binary classification problem. It proposes a handover technique that utilizes deep neural networks (DNNs). The handover strategy has two sets of neural networks that employ input criteria such as channel quality and user mobility for making intelligent decisions on handover. Following training with labeled datasets, the DNN-based handover approach achieves an accuracy rate exceeding 95%. Notably, the user throughput consistently outperforms that of the benchmark artificial neural network (ANN) for various users when employing the DNN, indicating a significant enhancement in the received signal strength of user equipment. Furthermore, the Quality of Service (QoS) with the DNN-based approach shows improvement. The DNN-based approach consistently achieves the highest user throughput, showcasing an improvement ranging from approximately 18.58% to 38.5% compared to the benchmark ANN. Additionally, the Cumulative Distribution Function (CDF) curves of the DNN-based approach for various user quantities exhibit the most variability, suggesting superior robustness performance compared to the benchmark ANN. This superiority is attributed to the consideration of multiple attributes in our approach for making handover decisions. Our research highlights the potential effectiveness of neural networks as a resilient solution for addressing handover challenges in heterogeneous networks. For future work, we need to focus on conducting an in-depth exploration into the computational complexity and processing time of the proposed method in comparison with state-of-the-art benchmark techniques. We posit that these findings can significantly contribute to future investigations into next-generation wireless networks, particularly those anticipated to feature ultra-dense configurations with line-of-sight communication links, such as millimeter-wave and LiFi networks.

## Figures and Tables

**Table 1:** WiFi/LiFi simulation parameters.

WiFi Simulation Parameters		LiFi Simulation Parameters	
Parameter	Value	Parameter	Value
Career Frequency	2.4 GHz	Room Size	(18 × 18 × 3) m^3^
Transmitted Power	20 dBm	No. of APs	36
Breaking-Point Distance	10 m	Semi Angle of Half Power	60°
Shadowing Fading St. Dev LOS/NLOS	3 dB/5 dB	Transmitted Power	9 W
Arrival/Departure Angle	45 degrees	Optical Gain	1
Noise PSD	−174 dBm/Hz	Noise PSD	10^−21^ A^2^/Hz
Bandwidth	20 MHz	Bandwidth	40 MHz
Modulation	64 QAM	Detector Responsivity	0.53 A/W

**Table 2:** Summarized handover accuracies and F1 scores of various schemes.

	Precision	Recall	Accuracy	F1 Score $=\frac{2\left(precision\;\times \;recall\right)}{presision\;+\;recall}$
K-LR	75.60%	82.12%	90.60%	0.79
K-SVM	77.70%	83.45%	91.40%	0.80
ANN	74.70%	65.25%	83.24%	0.70
FDDN	91.27%	91.28%	96.00%	0.91

## Data Availability

Data are contained within the article.
